# Dietary intake is associated with neuropsychological impairment in women with HIV

**DOI:** 10.1093/ajcn/nqab038

**Published:** 2021-04-07

**Authors:** Leah H Rubin, Deborah R Gustafson, Lakshmi Warrior, Lila Sheira, Kathryn C Fitzgerald, Raha Dastgheyb, Kathleen M Weber, Phyllis C Tien, Audrey French, Amanda B Spence, Anjali Sharma, Dionna W Williams, Cory J White, Eric C Seaberg, Edward A Frongillo, Sheri D Weiser

**Affiliations:** Department of Neurology, Johns Hopkins University School of Medicine, Baltimore, MD, USA; Department of Psychology, Johns Hopkins University School of Medicine, Baltimore, MD, USA; Department of Epidemiology, Johns Hopkins Bloomberg School of Public Health, Baltimore, MD, USA; Department of Neurology, State University of New York Downstate Health Sciences University, Brooklyn, NY, USA; Division of Neurology, Cook County Health, Chicago, IL, USA; Department of Medicine, Division of HIV, Infectious Diseases, and Global Medicine, University of California San Francisco, San Francisco, CA, USA; Department of Neurology, Johns Hopkins University School of Medicine, Baltimore, MD, USA; Department of Neurology, Johns Hopkins University School of Medicine, Baltimore, MD, USA; Division of Neurology, Cook County Health, Chicago, IL, USA; Cook County Health, Hektoen Institute of Medicine, Chicago, IL, USA; Department of Medicine, University of California, San Francisco, San Francisco, CA, USA; Department of Veterans Affairs Medical Center, San Francisco, CA, USA; Division of Neurology, Cook County Health, Chicago, IL, USA; Cook County Health, Hektoen Institute of Medicine, Chicago, IL, USA; Department of Medicine, Division of Infectious Disease and Travel Medicine, Georgetown University, Washington, DC, USA; Department of Medicine, Albert Einstein College of Medicine, Bronx, NY, USA; Department of Molecular and Comparative Pathobiology, Johns Hopkins University School of Medicine, Baltimore, MD, USA; Division of Clinical Pharmacology, Johns Hopkins University School of Medicine, Baltimore, MD, USA; Department of Biological Chemistry, Johns Hopkins University School of Medicine, Baltimore, MD, USA; Department of Epidemiology, Johns Hopkins Bloomberg School of Public Health, Baltimore, MD, USA; Department of Health Promotion, Education, and Behavior, University of South Carolina, Columbia, SC, USA; Department of Medicine, Division of HIV, Infectious Diseases, and Global Medicine, University of California San Francisco, San Francisco, CA, USA

**Keywords:** HIV, cohort studies, risk factors in epidemiology, neuropsychological assessment, diet, food, nutrition

## Abstract

**Background:**

Diet is a modifiable risk factor that may influence cognition in people with HIV.

**Objectives:**

We examined the association between dietary intake and cognition in women with HIV (WWH) and HIV-seronegative women.

**Methods:**

An 18-item dietary National Cancer Institute screener was completed by 729 WWH and 346 HIV-seronegative Women's Interagency HIV Study participants. Daily intake frequencies of processed meats, sweet beverages, fish, whole milk, and vegetables were calculated. Participants completed biennial neuropsychological (NP) testing. NP domains included attention/working memory, executive function, processing speed, memory, learning, fluency, and motor function. NP impairment was defined as demographically adjusted T-scores (mean = 50; SD = 10) ≤40 at ≥1 visit after completing the dietary screener. Multivariable logistic regression, stratified by HIV serostatus, examined associations between intake frequency tertile (referent = lowest intake) and NP performance.

**Results:**

Dietary intake frequencies of individual food line items were similar between WWH and HIV-seronegative women, except for sweet beverages, for which HIV-seronegative women reported higher intake frequencies than WWH (*P* values < 0.05). In WWH, multivariable-adjusted models indicated higher odds of NP impairment with higher intake frequencies of processed meat [*P *= 0.006; OR_upper tertile _= 1.91 (95% CI: 1.23–2.95; *P *= 0.003); OR_middle tertile _= 1.66 (95% CI: 1.14–2.42; *P *= 0.01)], sweet beverages [*P *= 0.02; OR_upper tertile _= 1.75 (95% CI: 1.17–2.64; *P *= 0.007)], fish [*P *= 0.01; OR_upper tertile _= 1.70 (95% CI: 1.10–2.64; *P *= 0.02)], and whole milk [*P *= 0.029; OR_upper tertile _= 1.66 (95% CI: 1.14–2.42; *P *= 0.008)]. Lower odds of NP impairment [*P *= 0.005; OR_upper tertile _= 0.65 (95% CI: 0.45–0.95; *P *= 0.02); OR_middle tertile _= 0.42 (95% CI: 0.24–0.73; *P *= 0.002)] were associated with higher vegetable intakes. In HIV-seronegative women, multivariable-adjusted models did not show associations between food line items/diet quality score and NP outcomes.

**Conclusions:**

Intakes of processed meat, sweet beverages, whole milk, fish, and vegetables may be associated with NP functions among WWH. Associations among WWH are not directly comparable to those among HIV-seronegative women, because models were conducted on each group separately given controls for HIV-specific covariates in WWH. Further studies are needed using more rigorous dietary assessment methods and lengthier longitudinal follow-ups.

## Introduction

Diet is associated with the development and prevention of late-onset Alzheimer's disease and related dementias (ADRD) (1, 2). Adherence to diets containing considerable amounts of vegetables, fruits, cereals, and fish; lower amounts of meat and saturated fats; and moderate amounts of alcohol are associated with lower prevalences and incidences of ADRD, milder cognitive impairment, lower conversion of mild cognitive impairment to Alzheimer's disease (AD) (3, 4), and prolonged survival among persons with AD (5). Mechanisms behind these observations involve inflammation, synaptic integrity, amyloid accumulation, and cellular metabolism and integrity, as well as endocrine and immune functions (2). While these observations are evident in typical aging populations, there is a dearth of information in underserved communities and in those with health disparities, notably including women with HIV (WWH).

Much of our knowledge about diet and chronic disease associations originates from cardiovascular disease and cancer epidemiology. From this literature, dietary screeners were created to rapidly assess dietary intake frequencies of key foods and/or food groups across communities based on public health recommendations for disease prevention (6, 7). Given similar dietary risks across aging-related outcomes, dietary screeners remain valuable for studies of neuropsychological (NP) performance, declines, and impairment, for which chronic diseases, such as cardiovascular disease, increase risks (2).

To understand dietary intakes in association with future NP performance in a sample of WWH and HIV-seronegative women, we tested dietary intake frequencies of key indicator foods or food groups, using a simple dietary screener developed by the US National Cancer Institute (6), in association with NP functions 2–4 y later in the Women's Interagency HIV Study (WIHS).

## Methods

The WIHS is a multicenter, prospective cohort study of women living with and without HIV, including those that enrolled initially in 1994–1995 at 6 sites (Bronx/Manhattan, NY; Brooklyn, NY; Chicago, IL; Washington, DC; San Francisco, CA; and Los Angeles, CA) and those that enrolled in 2001–2002 and 2011–2014. WIHS methods and baseline cohort characteristics have been described previously (8, 9). At semiannual visits, participants completed physical examinations, provided biological specimens, and underwent extensive clinical and behavioral assessments via face-to-face interviews. A comprehensive NP test battery was administered every 2 y beginning in 2009. The NP battery was always administered in the same order at a single visit by a single, certified interviewer, immediately following a phlebotomy and consumption of a snack by the participant. This occurred at the beginning of the visit, before any of the core interviews or examinations (physical or gynecological) had been administered. This ensured that women completing the NP battery were well rested, not hungry or thirsty, and in top mental condition for the testing. Administration of a single dietary screener occurred beginning in 2013 at the Bronx, Brooklyn, Chicago, DC, and San Francisco sites. This dietary screener administration was considered the baseline for analyses presented herein. Only NP test batteries conducted at or after implementation of the baseline dietary screener were considered in these analyses.

### Primary exposure variables

Dietary intake was measured using an adapted version of the Year 2000 National Health Interview Survey multifactor dietary screener (**Table 1**) (6). This dietary screener assessed the dietary intake frequencies as times per day, week, month, or year of 18 food line items, representing 50 foods or food groups. All intake frequencies were converted to daily intake frequencies. Missing values (<1%) were imputed under the assumption of missing at random to ensure complete cases. Finally, tertiles of daily intake frequencies were computed for each food line item, since they were not normally distributed.

**FIGURE 1:**
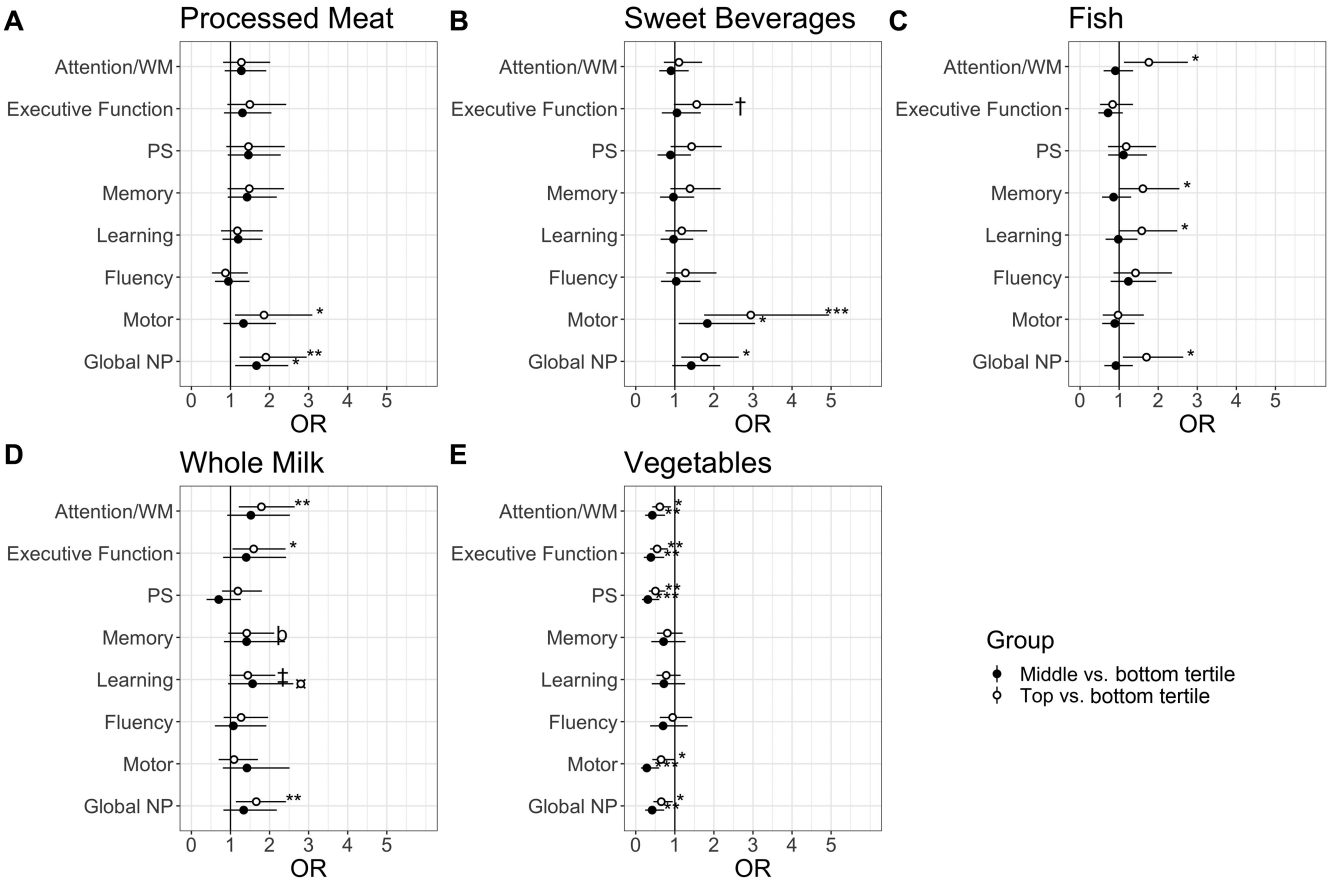
Dietary intake frequencies of (A) processed meats, (B) sweet beverages, (C) fish, (D) whole milk, and (E) vegetables and the odds of subsequent 2–4-year NP impairment among women with HIV (*n* = 729). *P < 0.05; **P < 0.01; ***P < 0.001; †*P *= 0.06; ‡*P *= 0.07; ¤ *P *= 0.08; þ *P *= 0.09. Multivariable logistic regression models were adjusted for: clinic site; annual household income; CES-D score ≥ 16; recent tobacco, marijuana, crack, cocaine, and/or heroin use; and HIV-specific clinical factors [history of any prior AIDS-defining illness, current CD4+ T-cell count (cells/mL), and years on ART]. Abbreviations: ART, antiretroviral therapy; CES-D, Center for Epidemiology Studies, Depression; NP, neuropsychological; PS, processing speed; WM, working memory.

**FIGURE 2:**
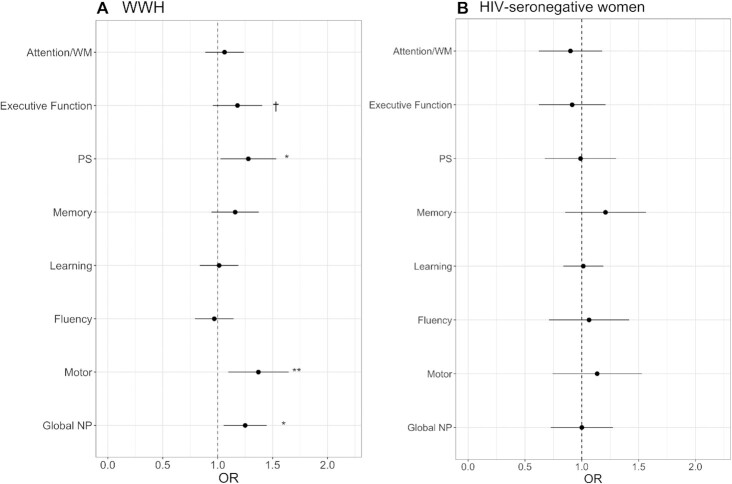
Diet quality score (higher score denoting less quality) and odds of subsequent 2–4-year NP impairment among (A) WWH (*n* = 729) and (B) HIV-seronegative women (*n* = 346). *P < 0.05; **P < 0.01; †*P *= 0.08. Multivariable logistic regression models were adjusted for: clinic site; annual household income; CES-D score ≥ 16; recent tobacco, marijuana, crack, cocaine, and/or heroin use; and, among WWH only, HIV-specific clinical factors [history of any prior AIDS-defining illness, current CD4+ T-cell count (cells/mL), and years on ART]. Abbreviations: ART, antiretroviral therapy; CES-D, Center for Epidemiology Studies, Depression; NP, neuropsychological; PS, processing speed; WM, working memory; WWH, women with HIV.

**TABLE 1:** Computation of a diet quality score based on individual line items comprising the Dietary Screener

	Score per tertile		
DS line item	Bottom	Middle	Top
Hot dogs, bacon, sausage, or lunch meats	1	2	3
Pork, beef, hamburgers, cheeseburgers, or meatloaf	1	2	3
Chicken, turkey, or duck	3	2	1
Beans, tofu, nuts, or lentils	3	2	1
Fish or seafood	3	2	1
Yogurt, cheese, cheese spreads, or cottage cheese	3	2	1
Doughnuts, cookies, cake, pastry, pies or chips	1	2	3
Nondiet sodas or fruit drinks	1	2	3
Cereal, rice, pasta, breads, tortillas, or other grains	3	2	1
Butter, margarine, full-fat salad dressing or mayonnaise	1	2	3
Eggs	1	2	3
Whole milk	1	2	3
Low-fat milk	3	2	1
100% fruit and tomato juices	3	2	1
Fruit	3	2	1
French fries or fried potatoes	1	2	3
Potatoes	3	2	1
Vegetables or green salad	3	2	1

The diet quality score is the sum of the scores, where a total score of 18 indicates the lowest and best dietary score and 54 indicates the highest and worst dietary score.

Based on the published literature in ADRD (10, 11), a diet quality score was created by assigning 1–3 points to each intake frequency tertile for each of the 18 food line items (Table 1), with 1 point assigned for what was deemed the highest quality food or food line item and 3 points assigned for what was deemed the lowest quality. For example, the highest intake frequency tertile of the vegetable food line item was assigned 1 point, whereas the highest intake frequency tertile of the processed meat food line item was assigned 3 points. There are no nutrient composition estimates accompanying the dietary screener, so these assigned values, while crude, were based on known nutrient composition estimates of the foods listed in each food line item, in line with published diet quality scores (10, 11).

### Primary outcome: NP performance

The NP test battery, administered every 2 y, included the Letter-Number Sequencing (LNS) test, Trail Making Test (TMT), Stroop Test (color word, word reading), Hopkins Verbal Learning Test–Revised (HVLT-R), Symbol Digit Modalities Test (SDMT), Controlled Oral Word Association Test (COWAT), Category Fluency Test (Animals), and Grooved Pegboard (GPEG) test. Up to 3 NP test batteries were available. Performance on these tests was used to assess the following 7 NP domains: *1*) attention/working memory (outcomes: total correct on LNS control and experimental conditions); *2*) executive function [outcomes: time to completion on TMT Part B and Stroop color-word (interference) trial]; *3*) processing speed (outcomes: total correct on SDMT, time to completion on Stroop word-reading trial); *4*) memory (outcome: HVLT-R delayed recall); *5*) learning (outcome: total learning across HVLT-R trials); *6*) fluency (outcomes: total correct on COWAT and Animals); and *7*) fine motor skills (outcomes: total time to completion for each hand on the GPEG).

All timed outcomes were log transformed to normalize distributions and were reverse scored so higher scores represented better performance. Demographically adjusted T-scores were derived for each outcome based on HIV-seronegative women (12, 13). These demographic factors include self-reported age, education, Wide Range Achievement Test score, race (African American vs. not), and ethnicity (Hispanic vs. not). T-scores were used to create domain scores and a global NP performance score, as done in previous WIHS and Male AIDS Cohort Study analyses (12, 14, 15). Next, these T-scores were used to categorize participants’ global NP and domain-specific profiles as 1 of the following 3 outcomes: *1*) NP healthy (T-score > 40 at all test battery time points); *2*) fluctuating (T-score ≤ 40 at ≥1 time point; i.e., in-and-out of NP impairment); or *3*) stably impaired (T-score ≤ 40 at all time points). The latter 2 profiles were considered as having an NP impairment. A 1 SD cutoff was selected for determining impairment, similar to previous NeuroHIV studies (12, 16).

### Covariates

Evaluated covariates included the WIHS clinic site and enrollment year (1994–1995, 2001–2002, 2011–2012) and self-reported sociodemographic, behavioral, and clinical factors. The socio-demographic covariates were age, self-reported years of education, race/ethnicity, and annual household income (≤$12,000). The behavioral covariates were current tobacco use; recent (last 6 months) heavy alcohol use (>7 drinks/week or ≥4 drinks in 1 sitting); and recent (last 6 months) marijuana, crack, cocaine, and/or heroin use. Clinical covariates were body weight (kg), body height (m), hepatitis C virus infection, clinically relevant depressive symptoms [Center for Epidemiology Studies, Depression (CES-D) score ≥ 16], systolic and diastolic blood pressures, and diabetes. Non–antiretroviral therapy (non-ART) medications with known adverse central nervous system (CNS) effects (17) linked to NP (14) were also examined, and included opioids, anticonvulsants, anticholinergics, antianxiety medications, antihistamines, antidepressants, gastrointestinal agents, beta-blockers, antipsychotics, and muscle relaxants. The number (0, 1, ≥2) of these non-ART CNS-active medications reported at the dietary screener visit were analyzed. Among WWH only, HIV-related clinical covariates were also considered, including current (i.e., at index visit) CD4+ count (<200, 200–500, >500 cells per mL), nadir CD4+ count (lowest CD4 measured in cells per mL prior to the index visit), current suppressed (<20 copies/mL) HIV-1 RNA, current combination ART use and adherence, years on ART, and history of prior AIDS-defining illness. There were 3 WWH missing data on current CD4+ count, 5 missing data on current suppressed HIV RNA, and 1 missing data on current ART use and adherence. Across all covariates, <1% of the data were missing.

### Ethics

Written informed consent was provided by each WIHS participant via human subject protocols that were approved by institutional review committees at each affiliated institution.

### Statistics

WIHS participants were characterized by serostatus on demographic and other descriptive factors and covariates using *t*-tests for continuous variables and chi-square tests for categorical variables. A series of chi-square tests stratified by HIV serostatus were computed to initially examine associations between frequency tertiles of dietary intake of each of the 18-dietary screener food line items and subsequent global NP and domain-specific NP impairment profiles. The highest and middle tertiles versus the lowest tertile of intake frequency were compared. Food line items significantly associated in univariate analyses with either global NP function or ≥2 NP domains were used for subsequent model consideration (**Supplemental Tables 1**and**2**) and were combined when possible into food groups to minimize the number of primary exposure variables. As a result, we combined food line items (≥1 food or food group per line) into the following 5 foods or food groups: processed meats (line items: hot dogs, bacon, sausage, or lunch meats), sweet beverages (line items: fruit juices, fruit drinks, or nondiet sodas), fish (line items: fish or seafood), whole milk, and vegetables (line items: vegetables or green salad).

Next, we conducted a series of multivariable logistic regression models, where we included each of the aforementioned 5 dietary screener foods or food groups as primary exposures of interest to examine associations with the described NP outcomes. The following potential confounders [listed under covariates and significantly (*P *< 0.10) relating to dietary intake and NP function] were also included: clinic site; annual household income ≤$12,000; CES-D score ≥ 16; recent tobacco, marijuana, crack, cocaine, and/or heroin use; and, among WWH only, HIV-specific clinical factors [current CD4+ T-cell count (cells/mL), years on ART, and history of any prior AIDS-defining illness].

To ensure that observed associations between the 5 derived foods or food group exposures and NP performance were not due to overall diet quality, a series of multivariable logistic regression analyses were conducted where we included the diet quality score in addition to the aforementioned potential confounders. The following statistical assumptions for logistic regression were examined and met: appropriate outcome structure (binary outcome), independent observations, absence of multicollinearity (exposure variables and covariates not too highly correlated with each other; examined via correlations; all *r* values < 0.29), linearity of exposure variables and log odds, and large sample size (least frequent outcome = 18%; 5 primary exposure variables; need 50/0.18 = 250 individuals) (18). ORs and 95% CIs were calculated using maximum likelihood estimates from the logistic regression models. Results were considered significant using 2-sided tests, and significance was set as a *P* value < 0.05. All analyses were performed using SAS (version 9.4, SAS Institute Inc.).

## Results

Dietary screeners and subsequent NP performance data were available for 1075 WIHS participants (729 WWH and 346 HIV-seronegative women; **Table 2**; **Supplemental Figure 1** for flow chart). NP performance was available at 2–3 time points (over a median 2 y of follow-up) after completion of the dietary screener. WWH were older (baseline age 49.1 vs. 47.5 y, respectively), more likely to be enrolled in earlier waves of the study, and more likely to identify as White and non-Hispanic compared to HIV-seronegative women (*P* values < 0.05). HIV-seronegative women were more likely to smoke; engage in heavy alcohol use; use marijuana, crack, cocaine, and/or heroin; and have hypertension (*P* values < 0.05).

**TABLE 2:** Demographics and other descriptive information about study participants by HIV serostatus

	HIV serostatus		
	HIV− (*n* = 346), *n* (%)	WWH (*n* = 729), *n* (%)	*P* value
Age, y, mean ± SD	47.5 ± 9.2	49.1 ± 8.4	0.004
Years of education, mean ± SD	12.3 ± 3.1	12.4 ± 3.0	0.80
WRAT-3 reading subtest, mean ± SD	89.7 ± 17.7	90.6 ± 18.2	0.45
BMI, kg/m^2^, mean ± SD	32.6 ± 9.2	30.4 ± 8.3	<0.001
Year of WIHS enrollment			0.004
1994–1995	131 (38)	350 (48)	
2001–2002	153 (44)	253 (35)	
2011–2014	62 (18)	126 (17)	
Number of NP visits completed			0.92
2 (baseline, 2 y)	341 (99)	719 (99)	
3 (baseline, 2 y, 4 y)	5 (1)	10 (1)	
Race			0.004
White, non-Hispanic	18 (5)	86 (12)	
Black, non-Hispanic	247 (71)	505 (69)	
Hispanic	64 (19)	105 (14)	
Other	17 (5)	33 (5)	
Annual income USD$ ≤ 12,000	174 (50)	362 (50)	0.84
Recent tobacco use^1^	172 (50)	273 (37)	<0.001
Heavy alcohol use^2^	83 (24)	93 (13)	<0.001
Recent marijuana use	101 (29)	146 (20)	<0.001
Recent crack, cocaine, and/or heroin use	39 (11)	55 (7)	0.04
Hepatitis C virus	49 (14)	137 (19)	0.06
Depression^3^	103 (30)	229 (31)	0.58
Use of CNS acting medications			0.19
None	212 (61)	419 (57)	
1	53 (15)	145 (20)	
2+	82 (23)	165 (22)	
Hypertension	181 (52)	320 (44)	0.009
Diabetes	78 (22)	162 (22)	0.91
HIV-specific factors			
CD4+ T-cell count, cells/mL			
<200	—	65 (9)	
200–499	—	219 (30)	
>500	—	442 (61)	
Nadir CD4+ T-cell count, cells/mL, median (IQR)	—	227 (259)	
Suppressed HIV-1 RNA, copies/mL	—	431 (59)	
cART and >95% adherent	—	524 (72)	
Years on ART, median (IQR)	—	14.2 (11.2)	
Years on cART, median (IQR)	—	12.7 (10.3)	
History of AIDS	—	267 (36)	

Data are presented as *n* (%) unless otherwise indicated. Variables reported as *n* (%) were analyzed with chi-square tests. Variables reported as mean ± SD were analyzed with independent t-tests. Abbreviations: ART, antiretroviral therapy; cART, combination antiretroviral therapy; CES-D, Center for Epidemiological Studies, Depression; CNS, central nervous system; HIV−, HIV seronegative; USD, US dollars; WIHS, Women's Interagency HIV Study; WRAT-3, Wide Range Achievement Test; WWH, women with HIV.

^1^Within 6 months of the most recent WIHS visit.

^2^Heavy alcohol use reflects >7 drinks/wk or ≥4 drinks in 1 sitting.

^3^Depression was calculated using CES-D scores ≥16.

There were similar proportions of WWH and HIV-seronegative women in the bottom, middle, and top tertiles of all food line items (*P* values > 0.05), except for sweet beverages, where HIV-seronegative women reported higher intake frequencies of both nondiet sodas or fruit drinks and 100% fruit juices compared to WWH (*P* values < 0.05; **Table 3**). With respect to NP profiles, there were higher proportions of WWH with fluctuating profiles on attention/working memory (*P *= 0.0002; **Table 4**) and executive function (*P *= 0.09) compared to HIV-seronegative women. There was also a higher percentage of WWH with a stable NP impairment profile on learning compared to HIV-seronegative women (*P *= 0.003). Given the small proportion of participants demonstrating a fluctuating profile on any of the outcomes, primary analyses were conducted using a combined group of stable NP impairment and fluctuating profiles.

**TABLE 3:** Percentage of HIV-seronegative women and women with HIV by dietary intake frequency tertile.

	% HIV− women^1^ (*n* = 346)			% WWH (*n* = 729)			
Dietary Screener line item	Bottom	Middle	Top	Bottom	Middle	Top	*P* value^2^
Hot dogs, bacon, sausage, or lunch meats	31	39	29	38	35	26	0.08
Pork, beef, hamburgers, cheeseburgers, or meatloaf	34	26	40	37	23	39	0.55
Chicken, turkey, or duck	40	26	34	36	31	33	0.21
Beans, tofu, nuts, or lentils	33	38	29	32	35	32	0.71
Fish or seafood	29	47	24	34	41	25	0.13
Yogurt, cheese, cheese spreads, or cottage cheese	29	35	35	32	34	34	0.69
Doughnuts, cookies, cake, pastry, pies, or chips	29	37	34	31	38	31	0.57
Nondiet sodas or fruit drinks	35	27	38	38	32	29	0.02
Cereal, rice, pasta, breads, tortillas, or other grains	40	11	48	37	15	48	0.30
Butter, margarine, full-fat salad dressing, or mayonnaise	29	32	39	26	39	35	0.07
Eggs	37	32	31	39	36	25	0.07
Whole milk	51	16	33	46	16	33	0.14
Low-fat milk	51	16	34	51	16	33	0.96
100% fruit and tomato juices	25	37	38	32	36	32	0.04
Fruit	34	23	43	31	22	46	0.63
French fries or fried potatoes	30	38	32	35	36	29	0.22
Potatoes	40	29	31	38	28	34	0.65
Vegetables or green salad	33	12	54	34	13	52	0.82
Diet quality score	29	36	35	37	30	33	0.04

Abbreviations: HIV−, HIV seronegative; WWH, women with HIV.

1 Percents may not total to 100 due to rounding errors.

2 *P* values refer to overall differences in proportions (using chi-square tests) between WWH and HIV-seronegative women.

**TABLE 4:** Percentage of HIV-seronegative women and women with HIV demonstrating a detrimental (stable impairment or fluctuating in-and-out of impairment) or healthy neuropsychological profile 2–4 y after dietary screener administration

	HIV− women (*n* = 346)			WWH (*n* = 729)			
Outcome	Healthy, *n* (%)	Stable impaired, *n* (%)	Fluctuate, *n* (%)	Healthy, *n* (%)	Stable impaired, *n* (%)	Fluctuate, *n* (%)	*P* value^1^
Attention/WM	265 (76)	68 (20)	13 (4)	493 (68)	157 (21)	79 (11)	0.0002
EF	277 (81)	48 (14)	21 (6)	542 (74)	120 (16)	67 (9)	0.09
PS	270 (78)	47 (13)	29 (8)	558 (76)	98 (13)	73 (10)	0.69
Memory	249 (72)	67 (20)	30 (9)	530 (73)	142 (19)	57 (8)	0.89
Learning	261 (75)	54 (16)	31 (9)	496 (68)	180 (25)	53 (7)	0.003
Fluency	278 (80)	51 (15)	17 (5)	576 (79)	103 (14)	50 (7)	0.46
Motor	284 (82)	42 (12)	20 (6)	568 (78)	107 (15)	54 (7)	0.28
Global	227 (66)	65 (19)	54 (16)	434 (59)	166 (23)	129 (18)	0.15

Abbreviations: EF, executive function; HIV−, HIV seronegative; PS, processing speed; WM, working memory; WWH, women with HIV.

^1^ *P* values refer to overall differences in proportions (using chi-square tests) between WWH and HIV-seronegative women.

### Dietary intake and global NP impairment among WWH and HIV-seronegative women

Among WWH, results of unadjusted chi-square tests, used to estimate associations between the food line item of interest and NP impairment, are included in **Table 5**. Dietary intake frequencies of processed meats, sweet beverages, fish, whole milk, and vegetables (all *P* values < 0.05 except whole milk, which had a *P* value of 0.08) were associated with global NP performance. Results of multivariable models are depicted in **Figure 1**A (processed meats), Figure 1B (sweet beverages), Figure 1C (fish), and Figure 1D (whole milk), and Figure 1E (vegetables). Higher dietary intake frequencies of processed meats [*P *= 0.006; OR_upper tertile _= 1.91 (95% CI: 1.23–2.95; *P *= 0.003); OR_middle tertile _= 1.66 (95% CI: 1.14–2.42; *P *= 0.01)], sweet beverages [*P *= 0.02; OR_upper tertile _= 1.75 (95% CI: 1.17–2.64; *P *= 0.007)], fish [*P *= 0.01; OR_upper tertile _= 1.70 (95% CI: 1.10–2.64; *P *= 0.02)], and whole milk [*P *= 0.029; OR_upper tertile _= 1.66 (95% CI: 1.14–2.42; *P *= 0.008)] were associated with greater odds of demonstrating a subsequent impaired NP profile. Conversely, a higher intake frequency of vegetables was associated with lower odds of demonstrating an NP impaired profile [*P *= 0.005; OR_upper tertile _= 0.65 (95% CI: 0.45–0.95; *P *= 0.02); OR_middle tertile _= 0.42 (95% CI: 0.24–0.73; *P *= 0.002)]. The diet quality score was associated with global NP function (*P *= 0.01). Lower diet quality, as indicated by a higher diet quality score, was associated with higher odds of demonstrating an NP impaired profile (OR = 1.25; 95% CI: 1.05–1.48; **Figure 2**A).

**TABLE 5:** Unadjusted associations between dietary intake frequency tertiles and percentages of HIV-seronegative women and women with HIV with subsequent neuropsychological impairments over 2–4 y

	Processed meats^1^				Sweet beverages^2^				Fish^3^				Whole milk^4^				Vegetables^5^			
	% Impaired				% Impaired				% Impaired				% Impaired				% Impaired			
Outcomes	B	M	T	*P* value	B	M	T	*P* value	B	M	T	*P* value	B	M	T	*P* value	B	M	T	*P* value
HIV− women																				
Attention/WM	30	20	22	0.18	26	22	23	0.74	23	24	24	0.99	24	24	22	0.87	28	28	19	0.11
EF	21	21	18	0.82	21	18	21	0.87	21	20	18	0.87	21	16	21	0.66	24	21	18	0.42
PS	27	19	20	0.30	21	22	22	0.98	20	23	22	0.86	20	20	25	0.51	28	19	19	0.16
Memory	33	23	30	0.22	25	28	30	0.77	22	33	27	0.20	29	23	30	0.59	29	35	26	0.45
Learning	30	19	27	0.15	27	24	23	0.74	19	27	26	0.37	25	18	29	0.24	26	32	21	0.25
Fluency	24	16	21	0.24	22	11	24	0.03^6^	19	23	15	0.28	19	19	20	0.96	26	19	16	0.16
Motor	14	16	26	0.06^7^	17	17	18	0.94	18	18	16	0.84	17	15	20	0.61	22	9	18	0.17
Global NP	37	32	36	0.68	35	31	36	0.73	29	39	32	0.25	31	28	42	0.07^7^	39	32	32	0.46^7^
WWH																				
Attention/WM	30	34	32	0.64	33	27	39	0.01^6^	31	29	41	0.02^6^	27	35	40	0.003^6^	39	24	30	0.01^6^
EF	21	26	31	0.05^7^	23	23	31	0.11	30	24	23	0.16	20	29	33	0.001^6^	34	16	22	<0.001^6^
PS	19	25	27	0.07^7^	21	22	29	0.07^7^	24	24	23	0.98	22	20	28	0.12	32	13	20	<0.001^6^
Memory	23	28	32	0.10	25	24	33	0.04^6^	27	23	34	0.04^6^	23	28	33	0.03^6^	30	24	26	0.43
Learning	30	34	33	0.69	31	30	35	0.48	31	29	38	0.17	29	36	35	0.16	36	29	30	0.30
Fluency	20	22	20	0.91	19	20	24	0.37	19	21	24	0.46	19	20	25	0.20	22	16	21	0.51
Motor	16	22	30	0.001^6^	13	23	31	<0.001^6^	23	22	21	0.85	20	27	26	0.02^6^	29	10	20	<0.001^6^
Global NP	33	43	47	0.005^6^	35	38	50	0.004^6^	39	37	47	*0.08*	34	40	50	<0.001^6^	48	30	39	0.007^6^

HIV− *n* = 346; WWH *n* = 729. Chi-square tests were used. Abbreviations: B, bottom tertile; EF, executive function; HIV−, HIV seronegative; M, middle tertile; NP, neuropsychological; PS, processing speed; T, top tertile; WM, working memory; WWH, women with HIV.

^1^Processed meats include hot dogs, bacon, sausage, or lunch meats.

^2^Sweet beverages include 100% fruit juices and nondiet sodas or fruit drinks.

^3^Fish includes fish or seafood.

^4^Whole milk includes drinks made with whole milk or whole milk on cereal.

^5^Vegetables include vegetables or green salad.

^6^ *P *< 0.05

^7^ *P *> 0.05 and *P *< 0.10.

Among HIV-seronegative women, results of chi-square tests to examine food line items are included in Table 5. None of the food line items (for processed meats, sweet beverages, fish, whole milk, or vegetables) were associated with global NP function (*P* values > 0.05). Results of the multivariable models mirrored the unadjusted results. The diet quality score was also not associated with global NP function in HIV-seronegative women (OR = 1.00; 95% CI: 0.76–1.33; *P *= 0.98; Figure 2B).

### Dietary intake and domain-specific NP impairment among WWH and HIV-seronegative women

Among WWH, results of the chi-square tests indicated that the dietary intake frequency of whole milk was associated with poorer performance across 4 NP domains: executive function, attention/working memory, memory, and motor function (*P* values < 0.05; Table 5). Intake frequency of vegetables was protective across 4 NP domains: executive function, attention/working memory, processing speed, and motor function (*P* values < 0.05). Intake frequencies of sweet beverages and fish were associated with poorer performance on attention/working memory and memory. Sweet beverages, as well as processed meats, were also associated with poorer motor functions (*P* values < 0.01). After a multivariable adjustment, higher intake frequencies of processed meats and sweet beverages were associated with higher odds of NP impairment on motor functions (Figure 1A–B). A higher intake frequency of fish and seafood was associated with higher odds of NP impairment on attention/working memory and learning and memory (Figure 1C). A higher intake frequency of whole milk was associated with higher odds of NP impairment on attention/working memory and executive function (Figure 1D). In contrast, a higher intake frequency of vegetables was associated with lower odds of NP impairment on attention/working memory, executive function, processing speed, and motor function (Figure 1E). The diet quality score was associated with motor function and processing speed only, such that a higher diet quality score (lower quality) was associated with higher odds of NP impairments on motor function (OR = 1.37; 95% CI: 1.11–1.69; *P *= 0.003) and processing speed (OR = 1.28; 95% CI: 1.05–1.56; *P *= 0.01; Figure 2).

Among HIV-seronegative women, results of the chi-square tests are included in Table 5. Only the intake frequency of sweet beverages was associated with fluency (*P *= 0.03), but this was driven by a difference between the top and middle tertiles. Processed meats, fish, whole milk, and vegetables were not associated with domain-specific NP functions. In multivariable models, none of the food or food groups, nor the diet quality score, was associated with a domain-specific NP function (*P* values > 0.20; Figure 2).

## Discussion

This prospective, multicenter cohort study suggests that dietary intake may be associated with NP impairments and/or declines among WWH compared to socio-demographically similar HIV-seronegative women. Higher intake frequencies of processed meats, sweet beverages, fish, and whole milk were associated with greater odds of global NP impairment among WWH, whereas a higher vegetable intake frequency was associated with lower odds. Certain foods or food groups were also associated with domain-specific NP impairments among WWH. For example, intake frequencies of processed meats and sweet beverages were associated with poorer motor function, whereas higher intake frequencies of fish and whole milk were associated with poorer performance of higher-order NP functions, including attention/working memory. It is unclear why associations were observed among WWH and not HIV-seronegative women. The metabolic aberrations that occur with HIV infection, as well as those occurring with ART, may make WWH more susceptible to NP impairments, in association with dietary exposures (19); however, further research is needed.

A higher intake frequency of processed meats may be detrimental to both NP performance and overall brain health. Processed meats are high in salt, fat, nitrates, and other preservatives; are energy dense (high kcal/g); and are poor in nutrients (20). They are readily accessible and easier to store compared to fresh meats due to salting, curing, fermentation, or smoking. Higher salt intake is a risk factor for hypertension (21), which is among the strongest and most consistent vascular risk factors for cognitive impairment, cognitive decline, and ADRD (22). Salt-sensitive hypertension is often common among those who are genetically susceptible and in certain race/ethnic groups, such as African Americans (23), who predominate in our sample.

“Sweet beverages” is a heterogeneous food group characterized by high amounts of simple sugars, and therefore energy, per unit volume (20, 24), and sweet beverages are consumed in high quantities (25, 26). High simple sugar consumption is a risk factor for obesity and type 2 diabetes (21), both known risk factors for NP impairment and ADRD (27). One of these added simple sugars, fructose, may accelerate the path to obesity and metabolic insults (28); this is further confounded by the definition of a serving in this era of “Big Gulps” and “Super Sizes.” To avoid multiple comparisons, we created a sweet beverages category that did not separate natural juices (fresh or prepared from frozen or liquid concentrate) from sodas or fruit drinks. Our dietary screener also does not include details on fortification with calcium or vitamin C, which would increase the nutrient density of these beverages (20).

Whether dairy consumption is beneficial or detrimental for brain health is controversial (29–31). Components of fluid milk (20), such as certain proteins [e.g., whey (32)], fatty acids, calcium, and vitamins A and D (29), are associated with better brain health. However, population-based longitudinal studies also report adverse associations of “dairy foods” on NP performance when considering dietary fat intake from dairy sources (29, 33). Our findings support this. While whole milk was adversely associated with NP performance, no associations were found for the dietary screener food lines of “yogurt, cheese, cheese spreads, or cottage cheese” or “butter, margarine, full-fat salad dressing, or mayonnaise.”

Higher intake of fish—particularly dark, fattier fish and the fatty acids concentrated in them, such as the omega-3 fatty acids EPA and DHA (20)—is associated with reduced risks of cognitive decline and dementia (33–36). Thus, fatty acids are added to FDA-approved nutritional products for cognition, such as Souvenaid (37, 38). The association of a higher intake frequency of “fish or seafood” with higher odds of NP impairment in this sample of middle-aged WWH is incongruent. Intake of fish or seafood, as queried on the dietary screener, may indicate intake of processed fish, such as frozen fish products (e.g., fish sticks) or fried fish sandwiches. Fish products are made from lower-quality fish, are battered and fried, contain higher proportions of saturated fatty acids, and are often served with tartar and other higher-fat and -sodium sauces. Reported fish intake may also indicate consumption of highly salted, dried fish, such as bacalao (20), which is common in urban Caribbean areas such as central Brooklyn. Thus, a variety of fish and seafood preparations are possible when interpreting the dietary screener line item “fish and seafood” (39). Similar distinctions in health benefits versus detriments are reported in the Reasons for Geographic and Racial Differences in Stroke Study (40).

“Vegetables or green salad” contain antioxidants, anti-inflammatory agents, fat-soluble vitamins, and lipids comprising neuronal cell membranes (2, 20, 41). The Atherosclerosis Risk in Communities study reported that higher plant food intake was associated with reductions in the cardiovascular disease incidence and overall mortality (42), and the Mediterranean dietary pattern is associated with milder cognitive impairment and AD (1–4). Vegetables have a lower energy density (4 kcal/g) compared to fats (9 kcal/g) or alcohol (7 kcal/g) (43) and are rich in dietary fiber, which contributes to better gastrointestinal health (20, 43). Vegetables are also not often consumed alone. Instead, they are frequently consumed with sauces that are high in fat (e.g., cheese) or sodium (e.g., table salt, soy sauce) (20). The importance of the bioactive “dose” of a plant food may override its preparation (44). From a public health perspective, campaigns such as “5 A Day for Better Health” (45) (referring to 5 servings of vegetables and fruits) and “Eat More Plant Foods” (46) recommend vegetable consumption without specifying preparation methods (22).

We found no association between several foods or food groups that the published literature suggests may influence NP performance. This could be due to the sample characteristics, including race/ethnicity, genetic diversity, sociodemographic underrepresentation, and health disparities. In addition, an 18-item dietary screener is inadequate to identify nuances related to preparation and other details, such as the type of fish. This is better assessed using more comprehensive dietary assessment protocols in larger samples of aging adults, such as the Nurses’ Health Study (45–47).

Our study has several strengths. First, this is an initial report examining brain health in association with dietary intake in >1000 WWH and HIV-seronegative women, among whom health disparities arise from socioeconomic, race/ethnicity, education, health-related, and other factors. The importance of exploring diet and brain health in communities with health disparities is illustrated by longitudinal data from both the National Health and Nutrition Examination Survey and the Behavioral Risk Factor Surveillance Systems in the United States. These studies show that race/ethnicity, education, and income disparities are associated with dietary intakes and intake patterns over time (48, 49), especially among adults age 50 y and older (50). Second, our participants are members of a well-characterized cohort who participate in routine NP performance assessments over time. Third, we used a dietary screener that was validated and developed by the National Cancer Institute (6) for use in similar communities. Finally, despite the lack of information about total dietary and energy intakes, there is precedent for using key indicator foods to examine diet quality (51, 52).

Our study also has limitations. First, dietary assessment was limited to 18 self-reported food line items, and *1*) higher intake frequencies were correlated with higher energy intake, for which there is no estimate; *2*) only intake frequency was queried; *3*) referent serving sizes were not provided; *4*) details of the foods comprising each line item are unknown; and *5*) nutrient content is not available. Second, how participants interpreted the dietary screener foods and food groups is unknown; thus, estimates are susceptible to reporting bias. However, cognitive interviewing has been accomplished using dietary screeners and an evidence base supports them (7). Third, participants in this study were younger compared to those participating in prospective studies of aging and NP function. Therefore, few participants were identified as having a clinically relevant NP impairment, and none had ADRD. Fourth, our findings cannot be generalized to men. Fifth, we did not adjust for multiple comparisons; however, we did create combination food groups as described and engaged in manual statistical model building to reduce this possibility. Sixth, the number of HIV-seronegative women was substantially smaller than the number of WWH. Finally, statistical models differed for WWH versus HIV-seronegative women, since additional HIV-related covariates were necessary for inclusion in the models for WWH.

To our knowledge, this is the first prospective study of dietary intake in association with NP functions in WWH. Our results do not imply that WWH and HIV-seronegative women differ with respect to the association of dietary intake on NP impairment. Results cannot be directly compared between WWH and HIV-seronegative women, since models for WWH included additional HIV-related covariates. Findings from this novel study contribute to an important foundation for future, more rigorous investigations of dietary intake–NP function associations in underrepresented WWH and HIV-uninfected women. Potential targets for intervention to prevent NP impairment in WWH can be identified, noting that HIV infection may exacerbate the health effects of certain foods.

## Supplementary Material

nqab038_Supplemental_File

## Data Availability

Data described in the manuscript, code book, and analytic code will be made available upon request pending Male AIDS Cohort Study (MACS)/ Women's Interagency HIV Study (WIHS) Combined Cohort Study (MWCCS) Executive Committee approval.

## Notes

The contents of this publication are solely the responsibility of the authors and do not represent the official views of the NIH.

Supplemental Tables 1 and 2 and Supplemental Figure 1 are available from the “Supplementary data” link in the online posting of the article and from the same link in the online table of contents at https://academic.oup.com/ajcn/.

LHR and DRG contributed equally to this work.

Abbreviations: AD, Alzheimer's disease; ADRD, Alzheimer's disease and related dementias; ART, antiretroviral therapy; CES-D, Center for Epidemiology Studies, Depression; CNS, central nervous system; COWAT, Controlled Oral Word Association Test; GPEG, Grooved Pegboard; HVLT-R, Hopkins Verbal Learning Test–Revised; LNS, Letter-Number Sequencing; NP, neuropsychological; SDMT, Symbol Digit Modalities Test; TMT, Trail Making Test; WIHS, Women's Interagency HIV Study; WWH, women with HIV.
