# Supplementary material for: Dietary intake is associated with neuropsychological impairment in women with HIV
Source: Am J Clin Nutr. 2021 Apr 7;114(1):378–89. doi: 10.1093/ajcn/nqab038 (PMC8246600; doi:10.1093/ajcn/nqab038)
Supplement: nqab038_Supplemental_File [file nqab038_supplemental_file.docx]

**On-line Supplementary Material**

**Dietary intake is associated with neuropsychological impairment in women with HIV**

**Rubin, Gustafson *et al.***

**Supplemental Table 1.** Unadjusted associations (using Chi-squares) between dietary screener line item frequencies (tertile), the diet quality score, and subsequent NP impairment (% impaired) over 2-4 years among women with HIV (WWH; n=729).

|  | **Attention/WM** | | |  | **Executive Function** | | |  | **PS** | | |  | **Memory** | | |
| --- | --- | --- | --- | --- | --- | --- | --- | --- | --- | --- | --- | --- | --- | --- | --- |
| **Dietary Screener Line Items** | **B** | **M** | **T** |  | **B** | **M** | **T** |  | **B** | **M** | **T** |  | **B** | **M** | **T** |
| Hot dogs, bacon, sausage, or lunch meats | 30 | 34 | 32 |  | *21* | *26* | *31* |  | *19* | *25* | *27* |  | 23 | 28 | 32 |
| Pork, beef, hamburgers, cheeseburgers, or meatloaf | 35 | 32 | 30 |  | 25 | 22 | 28 |  | 23 | 21 | 25 |  | 28 | 25 | 27 |
| Chicken, turkey, or duck | 32 | 30 | 35 |  | 26 | 21 | 29 |  | 27 | 21 | 22 |  | 28 | 23 | 31 |
| Beans, tofu, nuts, or lentils | 30 | 33 | 33 |  | 29 | 24 | 24 |  | *28* | *24* | *18* |  | 29 | 28 | 25 |
| Fish or seafood | **31** | **29** | **41** |  | 30 | 24 | 23 |  | 24 | 24 | 23 |  | **27** | **23** | **34** |
| Yogurt, cheese, cheese spreads, or cottage cheese | 31 | 33 | 33 |  | 28 | 24 | 25 |  | 24 | 25 | 21 |  | 29 | 28 | 26 |
| Doughnuts, cookies, cake, pastry, pies or chips | 34 | 28 | 35 |  | 26 | 24 | 26 |  | 27 | 21 | 23 |  | 27 | 26 | 29 |
| Non-diet sodas or fruit drinks | 31 | 34 | 33 |  | **20** | **26** | **32** |  | 20 | 25 | 27 |  | 23 | 28 | 31 |
| Cereal, rice, pasta, breads, tortillas or other grains | 31 | 30 | 35 |  | 22 | 23 | 29 |  | 23 | 20 | 24 |  | 28 | 20 | 29 |
| Butter, margarine, full-fat salad dressing or mayonnaise | 38 | 29 | 32 |  | 26 | 23 | 28 |  | 26 | 22 | 23 |  | 29 | 26 | 28 |
| Eggs | 31 | 34 | 33 |  | 23 | 28 | 26 |  | 22 | 26 | 23 |  | 26 | 26 | 30 |
| Whole milk | **27** | **35** | **40** |  | **20** | **29** | **33** |  | 22 | 20 | 28 |  | **23** | **28** | **33** |
| Low-fat milk | 32 | 35 | 33 |  | 26 | 25 | 26 |  | 24 | 21 | 24 |  | 27 | 26 | 28 |
| 100% fruit juices | 28 | 34 | 36 |  | 24 | 24 | 28 |  | 22 | 22 | 28 |  | **23** | **26** | **33** |
| Fruit | 33 | 29 | 36 |  | 30 | 20 | 25 |  | 26 | 20 | 24 |  | 31 | 24 | 26 |
| French fries or fried potatoes | 32 | 29 | 36 |  | 25 | 22 | 29 |  | 24 | 23 | 23 |  | 29 | 27 | 25 |
| Potatoes | 30 | 33 | 34 |  | 24 | 27 | 25 |  | 25 | 21 | 25 |  | 27 | 27 | 27 |
| Vegetables or green salad | **39** | **24** | **30** |  | **34** | **16** | **22** |  | **32** | **13** | **20** |  | 30 | 24 | 26 |
| Diet quality score | 31 | 32 | 33 |  | **21** | **24** | **31** |  | **21** | **19** | **30** |  | *22* | *29* | *31* |

|  |  |  |  |  |  |  |  |  |  |  |  |  |  |  |  |
| --- | --- | --- | --- | --- | --- | --- | --- | --- | --- | --- | --- | --- | --- | --- | --- |
|  |  |  |  |  |  |  |  |  |  |  |  |  |  |  |  |
|  | **Learning** | | |  | **Motor** | | |  | **Fluency** | | |  | **Global NP** | | |
| **Dietary Screener Line Items** | **B** | **M** | **T** |  | **B** | **M** | **T** |  | **B** | **M** | **T** |  | **B** | **M** | **T** |
| Hot dogs, bacon, sausage, or lunch meats | 30 | 34 | 33 |  | **16** | **22** | **30** |  | 20 | 22 | 20 |  | **33** | **43** | **47** |
| Pork, beef, hamburgers, cheeseburgers, or meatloaf | 35 | 30 | 31 |  | 20 | 25 | 22 |  | 21 | 25 | 19 |  | 39 | 43 | 41 |
| Chicken, turkey, or duck | *31* | *28* | *37* |  | 26 | 20 | 20 |  | 22 | 18 | 24 |  | 41 | 37 | 43 |
| Beans, tofu, nuts, or lentils | 27 | 34 | 35 |  | **26** | **22** | **16** |  | 22 | 20 | 20 |  | 42 | 41 | 39 |
| Fish or seafood | 31 | 29 | 38 |  | 23 | 22 | 21 |  | 19 | 21 | 24 |  | *39* | *37* | *47* |
| Yogurt, cheese, cheese spreads, or cottage cheese | **39** | **27** | **31** |  | 26 | 22 | 19 |  | 22 | 20 | 21 |  | 46 | 40 | 38 |
| Doughnuts, cookies, cake, pastry, pies or chips | 34 | 32 | 30 |  | 21 | 21 | 25 |  | 25 | 19 | 19 |  | 43 | 36 | 43 |
| Non-diet sodas or fruit drinks | 31 | 35 | 31 |  | **13** | **25** | **31** |  | 22 | 19 | 23 |  | *35* | *42* | *45* |
| Cereal, rice, pasta, breads, tortillas or other grains | 33 | 26 | 33 |  | **27** | **14** | **21** |  | 21 | 16 | 22 |  | 40 | 36 | 42 |
| Butter, margarine, full-fat salad dressing or mayonnaise | 33 | 32 | 31 |  | 20 | 21 | 25 |  | 23 | 21 | 19 |  | 44 | 37 | 42 |
| Eggs | 29 | 34 | 33 |  | 20 | 26 | 19 |  | 21 | 21 | 21 |  | 37 | 45 | 41 |
| Whole milk | 29 | 36 | 35 |  | **18** | **27** | **26** |  | 19 | 20 | 25 |  | **34** | **40** | **50** |
| Low-fat milk | 34 | 30 | 30 |  | 23 | 15 | 24 |  | 21 | 18 | 23 |  | 41 | 41 | 40 |
| 100% fruit juices | 29 | 31 | 36 |  | 19 | 22 | 26 |  | 18 | 21 | 23 |  | **38** | **39** | **46** |
| Fruit | 34 | 28 | 32 |  | 23 | 19 | 23 |  | 20 | 19 | 22 |  | 44 | 38 | 39 |
| French fries or fried potatoes | *37* | *27* | *32* |  | *18* | *21* | *28* |  | 24 | 20 | 17 |  | 40 | 39 | 44 |
| Potatoes | 34 | 28 | 34 |  | 19 | 21 | 26 |  | 24 | 20 | 19 |  | 41 | 40 | 41 |
| Vegetables or green salad | 36 | 29 | 30 |  | **29** | **10** | **20** |  | 22 | 16 | 21 |  | **48** | **30** | **39** |
| Diet quality score | 29 | 33 | 34 |  | **16** | **21** | **30** |  | 21 | 19 | 22 |  | **36** | **36** | **49** |
|  |  |  |  |  |  |  |  |  |  |  |  |  |  |  |  |

Note. B=Bottom tertile; M=middle tertile; T=top tertile; NP=neuropsychological; WM=working memory; PS=processing speed; Bolded text denotes significant associations between dietary screener item or score and NP outcome at *P*<0.05 and italicized text denoted associations at *P*<0.10.

**Supplemental Table 2.** Unadjusted associations (using Chi-squares) between dietary screener line item frequencies (tertile), the diet quality score, and subsequent NP impairment (% impaired) over 2-4 years among HIV-seronegative women (n=346).

|  | **Attention/WM** | | |  | **Executive Function** | | | |  | | **PS** | | | |  | | **Memory** | | | |  |
| --- | --- | --- | --- | --- | --- | --- | --- | --- | --- | --- | --- | --- | --- | --- | --- | --- | --- | --- | --- | --- | --- |
| **Dietary Screener Line Items** | **B** | **M** | **T** |  | | **B** | **M** | **T** | |  | | **B** | **M** | **T** | |  | | **B** | **M** | **T** | |
| Hot dogs, bacon, sausage, or lunch meats | 30 | 20 | 22 |  | | 21 | 21 | 18 | |  | | 27 | 19 | 19 | |  | | 33 | 23 | 31 | |
| Pork, beef, hamburgers, cheeseburgers, or meatloaf | 29 | 21 | 20 |  | | 18 | 24 | 19 | |  | | 21 | 25 | 21 | |  | | 31 | 24 | 29 | |
| Chicken, turkey, or duck | 20 | 26 | 24 |  | | 21 | 20 | 18 | |  | | 22 | 20 | 22 | |  | | 30 | 25 | 27 | |
| Beans, tofu, nuts, or lentils | 27 | 20 | 23 |  | | **17** | **15** | **30** | |  | | 21 | 17 | 28 | |  | | 33 | 24 | 30 | |
| Fish or seafood | 23 | 24 | 24 |  | | 21 | 20 | 18 | |  | | 20 | 23 | 22 | |  | | 22 | 33 | 27 | |
| Yogurt, cheese, cheese spreads, or cottage cheese | 28 | 24 | 20 |  | | 22 | 20 | 19 | |  | | 24 | 23 | 19 | |  | | 30 | 32 | 23 | |
| Doughnuts, cookies, cake, pastry, pies or chips | 27 | 24 | 19 |  | | 17 | 25 | 16 | |  | | 21 | 27 | 17 | |  | | 33 | 27 | 24 | |
| Non-diet sodas or fruit drinks | 26 | 25 | 21 |  | | 21 | 17 | 22 | |  | | 22 | 22 | 22 | |  | | 27 | 28 | 29 | |
| Cereal, rice, pasta, breads, tortillas or other grains | 25 | 22 | 23 |  | | 23 | 15 | 18 | |  | | 23 | 15 | 23 | |  | | 30 | 17 | 29 | |
| Butter, margarine, full-fat salad dressing or mayonnaise | 27 | 24 | 20 |  | | *19* | *27* | *15* | |  | | 17 | 27 | 21 | |  | | 28 | 26 | 30 | |
| Eggs | 26 | 22 | 23 |  | | 23 | 19 | 18 | |  | | 25 | 19 | 22 | |  | | 27 | 27 | 32 | |
| Whole milk | 24 | 25 | 22 |  | | 21 | 16 | 21 | |  | | 20 | 20 | 25 | |  | | 29 | 24 | 30 | |
| Low-fat milk | 20 | 26 | 28 |  | | 21 | 20 | 18 | |  | | 23 | 13 | 24 | |  | | 28 | 22 | 32 | |
| 100% fruit juices | 23 | 24 | 24 |  | | 23 | 17 | 21 | |  | | 19 | 23 | 22 | |  | | *19* | *28* | *33* | |
| Fruit | 27 | 20 | 22 |  | | 21 | 16 | 21 | |  | | 27 | 18 | 20 | |  | | 31 | 28 | 26 | |
| French fries or fried potatoes | 24 | 27 | 20 |  | | 23 | 19 | 19 | |  | | 24 | 24 | 17 | |  | | 28 | 25 | 33 | |
| Potatoes | 22 | 27 | 22 |  | | 19 | 17 | 24 | |  | | 19 | 26 | 23 | |  | | 30 | 30 | 23 | |
| Vegetables or green salad | 28 | 28 | 19 |  | | 24 | 21 | 18 | |  | | 28 | 19 | 19 | |  | | 29 | 35 | 26 | |
| Diet quality score | 25 | 21 | 24 |  | | 17 | 23 | 19 | |  | | 18 | 23 | 24 | |  | | 21 | 30 | 32 | |
|  |  |  |  |  | |  |  |  | |  | |  |  |  | |  | |  |  |  | |
|  |  |  |  |  | |  |  |  | |  | |  |  |  | |  | |  |  |  | |

|  |  |  |  |  |  |  |  |  |  |  |  |  |  |  |  |
| --- | --- | --- | --- | --- | --- | --- | --- | --- | --- | --- | --- | --- | --- | --- | --- |
|  | **Learning** | | |  | **Motor** | | |  | **Fluency** | | |  | **Global NP** | | |
| **Dietary Screener Line Items** | **B** | **M** | **T** |  | **B** | **M** | **T** |  | **B** | **M** | **T** |  | **B** | **M** | **T** |
| Hot dogs, bacon, sausage, or lunch meats | 30 | 19 | 27 |  | *14* | *16* | *26* |  | 24 | 16 | 21 |  | 37 | 32 | 36 |
| Pork, beef, hamburgers, cheeseburgers, or meatloaf | 28 | 20 | 25 |  | **14** | **27** | **16** |  | 18 | 23 | 19 |  | 36 | 39 | 31 |
| Chicken, turkey, or duck | 22 | 23 | 27 |  | 20 | 12 | 19 |  | 19 | 22 | 17 |  | 34 | 33 | 35 |
| Beans, tofu, nuts, or lentils | 28 | 20 | 28 |  | 18 | 19 | 17 |  | 24 | 15 | 20 |  | *39* | *27* | *40* |
| Fish or seafood | 19 | 27 | 26 |  | 18 | 19 | 16 |  | 19 | 23 | 15 |  | 29 | 39 | 32 |
| Yogurt, cheese, cheese spreads, or cottage cheese | 27 | 27 | 20 |  | 20 | 16 | 17 |  | 17 | 20 | 21 |  | 36 | 34 | 34 |
| Doughnuts, cookies, cake, pastry, pies or chips | 26 | 27 | 19 |  | 19 | 18 | 16 |  | 19 | 22 | 17 |  | 37 | 38 | 27 |
| Non-diet sodas or fruit drinks | 25 | 29 | 21 |  | 13 | 20 | 20 |  | 17 | 19 | 22 |  | 35 | 32 | 35 |
| Cereal, rice, pasta, breads, tortillas or other grains | 27 | 17 | 24 |  | 18 | 15 | 18 |  | 18 | 17 | 21 |  | *38* | *20* | *34* |
| Butter, margarine, full-fat salad dressing or mayonnaise | 23 | 25 | 25 |  | 15 | 23 | 16 |  | 17 | 21 | 20 |  | 34 | 34 | 34 |
| Eggs | 23 | 27 | 25 |  | 19 | 16 | 19 |  | 24 | 16 | 18 |  | 38 | 30 | 36 |
| Whole milk | 25 | 18 | 29 |  | 17 | 15 | 20 |  | 19 | 19 | 20 |  | *31* | *28* | *42* |
| Low-fat milk | 25 | 22 | 25 |  | 20 | 15 | 16 |  | 20 | 11 | 22 |  | 35 | 28 | 37 |
| 100% fruit juices | 23 | 21 | 30 |  | 17 | 18 | 18 |  | *19* | *13* | *25* |  | 29 | 32 | 40 |
| Fruit | 26 | 25 | 23 |  | 23 | 14 | 16 |  | 19 | 21 | 19 |  | 38 | 30 | 33 |
| French fries or fried potatoes | 22 | 26 | 25 |  | 18 | 16 | 21 |  | 20 | 22 | 17 |  | 38 | 35 | 31 |
| Potatoes | 26 | 28 | 19 |  | 19 | 18 | 16 |  | 24 | 17 | 17 |  | 33 | 38 | 32 |
| Vegetables or green salad | 26 | 32 | 21 |  | 22 | 9 | 18 |  | 26 | 19 | 17 |  | 39 | 32 | 32 |
| Diet quality score | 21 | 24 | 27 |  | 14 | 16 | 23 |  | 16 | 21 | 22 |  | 28 | 37 | 27 |

Note. B=Bottom tertile; M=middle tertile; T=top tertile; NP=neuropsychological; WM=working memory; PS=processing speed;

Bolded text denotes significant associations between dietary screener item or score and NP outcome at *P*<0.05 and italicized text denoted associations at *P*<0.10.

**Supplemental Figure 1.** Flow chart for the selection of the analytic sample.

**
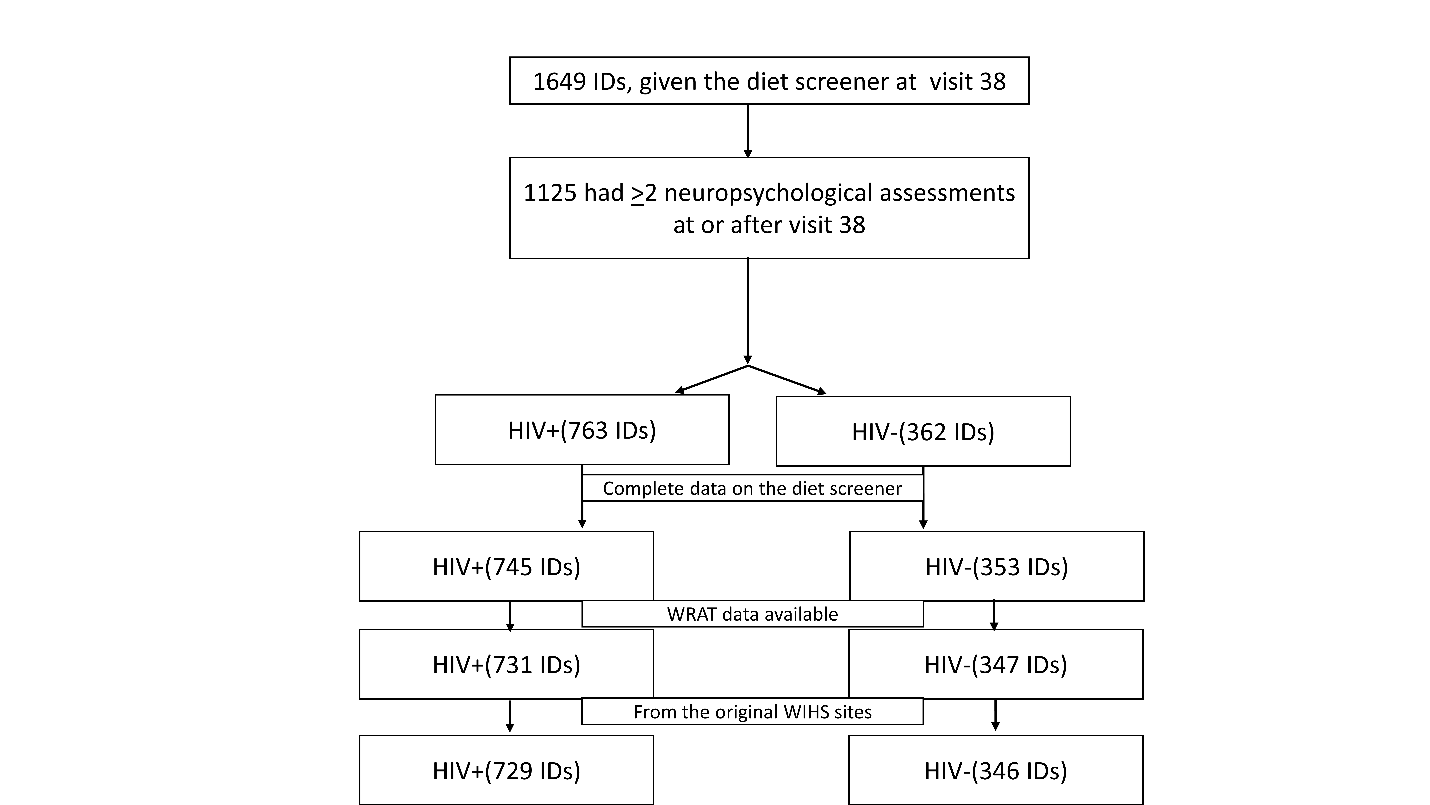
**
